# ‘GETTING’ THE POX: Reflections by an Historian on How to Write the History of Early Modern Disease

**DOI:** 10.5324/njsts.v2i1.2137

**Published:** 2014

**Authors:** Claudia Stein

**Keywords:** medical history, medical semiotics, constructivism

## Abstract

This article reflects upon the recent return to linear history writing in medical history. It takes as its starting point a critique of the current return to constructivist ideas, suggesting the use of other methodological choices and interpretations to the surviving archival and textural sources of the sixteenth century pox. My investigation analyses the diagnostic act as an effort to bring together a study of medical semiotics. Medical semiotics considers how signs speak through the physical body, coached within a particular epistemology. There are no hidden meanings behind the visible sign or symptom - it is tranparent to the calculative and authoritative gaze and language of the doctor. It concerns how diseases came into being, the relationships they have constituted, the power they have secured and the actual knowledge/power they have eclipsed or are eclipsing. From such a perspective, “getting the pox” is not a bad thing. A methodological turn to medical semiotics reminds us that the history of disease should be an inquiry both into the grounds of our current knowledge and beliefs about disease and how they inspire our writing, as well as the analytical categories that establish their inevitability.

## Introduction

Something strange has happened in the history of disease. Not long ago, when socio-cultural representations were the rage, disease was the laboratory for all kinds of application of constructionist ideas. But recently the laboratory has closed. Today in the writing on disease we witness a silent return to the empirical, the material, and the ‘real’. As Sander Gilman has observed, the study of health and disease as a time and space-specific representation has lost its allure, especially in Anglo-American scholarship. Indeed, the approach is increasingly disqualified as ‘merely’ the interest of a subjective history with no potential for more universal and transcendental meanings (Gilman 2011). Universal meanings or ‘lessons’ to be learned from the past are what now ‘sells’; the premises of theories of representation do not. Many historians of disease, inspired by the new ‘objective’ technologies of medical science, such as genetic engineering and brain scanning, are at the forefront of this new move to ‘the real.’ A telling example is the multi-volume Biographies of Disease, written and edited by eminent historians of medicine.^1^ In their stories of cholera, diabetes, asthma and so on, from ancient Greece to modern times, they embrace, wittingly or unwittingly, the old and worn method of retrospective diagnosis (Tattersall 2009, Jackson 2009, Hamlin 2009). We are back to linear stories of origin and continuity that the first generation of professional medical historians at the turn of the last century were so fond of, and through which they celebrated medical progress and ingenious doctors.

Like Gilman, I am disconcerted by this return, which seems ill-fitted to our post-postmodern times. Or, is it precisely because of our post-postmodern way of life that such histories of continuity and origin have new appeal? Could it be that they offer solace and a feeling of control and security in a world that fetishizes fluidity and constant change? Recently, in publications written with the historian of science and medicine Roger Cooter, I explored the possible reasons for this new empiricism and the problems it raises (Cooter and Stein 2013). Here I want to turn away from the methodological challenges of the present to reflect more on those of the past. Drawing on my presentation for the ‘Writing Nature in the History of Medicine’ lecture series at Oslo University in May 2013, I want to take this opportunity to re-engage with my earlier work on the history of the sixteenth-century pox to ask what methodological aims I was then pursuing, and why. As a social historian working on the history of disease in the 1990s, why was I so exited and challenged by constructionist ideas? And how did those ideas ultimately shape my choice and interpretation of the surviving archival and textual sources on the pox? At the end of this walk down memory lane I want briefly to return to the present, to reflect on whether constructionist ideas in the history of disease ought now to be abandoned in the light of the new essentialist claims.

## What is the French Pox?

When I first encountered constructionist ideas of disease I was working on the history of an epidemic that spread like a wildfire across Europe at the end of the fifteenth century. Like the Black Death roughly 150 years before, this new epidemic took its victims by surprise and quickly turned them into rotting piles of flesh (Stein 2009). Contemporary German-speaking authors who commented on the epidemic had no doubt about who was to blame. They traced it back to a specific historical event: the invasion of the Italian peninsula in 1494 by the armies of the French king, Charles VIII (1483-1498). They believed that the disease (a divine punishment of course) first erupted among Charles’ mercenaries, who at the cessation of the hostilities, returned to their respective homelands, thus spreading the new plague throughout Europe. In the German lands the disease was therefore labelled the French pox (*Franzosenpocken*), the French disease (*Franzosenkrankheit*) or, in Latin, *morbus gallicus*.^2^

Blaming someone other than God for the epidemic was one thing: but it was another to know how to confront it. Learned treatises in both Latin and German soon proliferated, presenting cosmological and physiological interpretations, and offering various therapies. But the confrontation with the pox was not confined to the world of letters. As ever-greater numbers of despairing victims hammered at the doors of civic charitable institutions, begging for help, the southern imperial city of Augsburg, one of Germany’s main trading centres at the time, was one of the first communities to practically respond to the new threat. In 1495, the town converted an old plague house into a civic pox hospital. This was then followed by the opening of two private hospitals in the city in 1523/24 and 1572, financed by members of the richest merchant-banker family in Europe at the time, the Fuggers.^3^ Treatment in all three hospitals was free.

What struck me as odd was that while the early modern medical literature and the surviving hospital records in Augsburg demonstrated that contemporaries struggled to come to terms with the nature of this unknown disease, the secondary literature barely recognised this. Instead the secondary literature identified the disease as venereal syphilis and confidently constructed its narrative around this biological ‘fact’, reducing it to one that focuses only on sexuality (Cooter 2013, Stein 2009).^4^ The belief that the French pox was in fact the sexually transmittable disease entity of venereal syphilis (a view that became hegemonic around the turn of the last century when the causal agent of venereal disease was first isolated in 1905 by the two German physicians Erich Hoffmann and Fritz Schaudinn) led historians to turn their attention primarily to archival and textual evidence that allegedly demonstrated the impact of the pox on sexual behaviour and moral attitudes of early modern European society.

This perspective fitted well with the widely accepted thesis at the time that proclaimed the ‘birth’ of the modern civilized individual during the Renaissance. Historians of syphilis argued that this process of individualisation was accelerated by the sudden appearance of venereal disease on European shores (Burckhardt 1990 [1878], Bloch 1901, Bloch and Loewenstein 1912). While sexual activity had been a matter of little restraint during the Middle Ages, they argued, the sudden arrival of syphilis made sixteenth-century contemporaries much more suspicious and cautious about the pleasures of the flesh. The closure of municipal brothels, the stigmatisation of prostitutes, and the abolition of the public bath culture – which are indeed reported in sixteenth-century European sources – were interpreted as direct responses to the sudden appearance of the French pox and its alleged influence on individual and collective human sexual behaviour, morals and values.

I have shown elsewhere how this ‘sex-focused’ selection and interpretation of sixteenth-century source materials on the pox was deeply shaped by late nineteenth- early twentieth-century concerns about sexually transmitted diseases which preoccupied not only the minds of the new medical elite of laboratory bacteriologists but also politicians and the public at large (Stein 2009, Sauerteig 1999). In Germany, for example, the discussion of the ‘Lustseuche’ (lust disease) as venereal syphilis was then named, and its perceived threat to the individual, the family, the state and the German race, was one of the central themes of social and political policies. Debates raged not only in the scientific community over its biological identity, but also, among the wider public.

The social roots of the disease, its dissemination, and how to measure it were all widely discussed with, ultimately, the regulation of prostitutes (the alleged chief propagators of the ‘sexual vice’) becoming the consensual solution. Syphilis hysteria was not peculiar to Germany. During the last decade of the nineteenth century doctors and lawyers, administrators, diplomats, church leaders, representatives of ethical and humanitarian movements, and women’s organisations from all over the world met at a series of international conferences to collaborate on strategies to solve the acute problems posed by syphilis and prostitution. The many contemporary ‘histories of syphilis’ written by medical practitioners (some of them bestsellers) offered a historical dimension to the perceived threat. Attention was paid to the origin of venereal syphilis and to different socio-cultural reactions to it throughout the centuries. The power of such histories of syphilis only ceased after World War II when the widespread introduction of antibiotics quieted fears over sex and disease.

With the advent of Aids in the 1980s, however, many of these deep-seated anxieties were rekindled, along with the old historical narratives. Perceived as primarily sexually transmitted, Aids initially challenged the post-war success story of bacteriology – indeed it put the whole bacteriological paradigm of Western medical science into question (Wolff 2012). Accompanying this was the rise of new ‘histories of syphilis’ and other sexually transmissible diseases, most of which simply repeated the old well-known old stories. As one author put it,

Then came the shock, at the moment when the Renaissance was beginning to unfold its petals into full bloom. The epidemic proportions of the new plague and the virulence of its effects turned the promiscuous habits of the time into a mortal danger. The bath houses were the first to suffer, and their closure was followed by restrictive measures directed against prostitutes and brothels in all cities of Europe’. (Fabricius 1994:17)

In retrospect I believe that my own interest in the pox was also initially sparked by the arrival of Aids and the challenges it presented to Western medicine. But the ‘re-emerging’ of such worn narratives nevertheless troubled me. This was because since the 1990s the studies of early modern sexuality and prostitution that I had drawn on in my research had convincingly refuted these myths, demonstrating that the closure of bathhouses and brothels had little to do with the new disease, but in fact more to do with the increasing economic difficulties that beset brothel-owners, cirumstances intimately linked to the propagation and implementation of new and stricter moral standards introduced with the Protestant Reformation (Roper 1991, Schuster 1992).

## Histories of disease

However, the new ‘histories of syphilis’ in the wake of Aids stubbornly disregarded this scholarship -- the scholarship that I was then poring my heart into (see for example Quetel 1992). But even more interesting to me was their refusal to engage with major challenges in historical methodology at the time. I myself was then working through Foucault’s ideas (Foucault 1973, 1989) and engaging with ideas debated in sociology, social science studies, and the history and philosophy of science and technology that problematised the production of scientific knowledge. The proposition of this work was that the experience of disease, its recognition and description was indivisible from the practices and logic of its treatment and institutionalisation – in short, that it was socially constructed. This was useful and challenging to think with, although I was probably not the only one bewildered by the great variety of views how exactly disease was supposed to be constructed (Hacking 2000). I also liked the political work these scholars were doing when they exposed the silent epistemological assumptions, hidden behind allegedly ‘objective’ knowledge-producing practices about disease such as laboratory experimentalism.

I began to wonder why historians of medicine, particularly those working on past diseases such as the pox, hesitated seriously to engage with the core ideas of social constructionism. Over the course of my research I gathered that this avoidance had to do with long-standing intellectual traditions in the history of medicine, which had been approaching the question of disease in history rather differently. One of the oldest and strongest of these traditions, reaching back to the turn of the last century when the history of medicine turned into an academic discipline, investigated disease from the perspective of public health. These historians therefore evaluated the impact of disease on individuals and populations. State policy was therefore their central point of reference. It is not difficult to see how the arrival of Aids revitalised this form of history writing on disease, which relied upon contemporary policy debate.^5^ Tracing individual and social reactions to specific diseasea over time, such narratives did not problematise the very object of their investigation, the disease itself. Rather, they treated it as a transhistorical and a stable category, while the socio-cultural reactions to it were represented as being in flux.

I detected a similar take on the history of disease among social and cultural historians of medicine, another strong tradition in this field of scholarship (Cooter 2006). Social history of medicine, originating in the 1930s but strengthened in the 60s and 70s through a leftist political agenda that critiqued and questioned power relationships in medicine, turned away from the progress stories of doctors and scientists. Its practitioners embraced ‘a history from below’, choosing heroes who had previously remained silent in medical history writing – notably patients, particularly women, that mad or the poor. But their narratives strongly focussed on the social reactions to disease: how knowledge about the disease under investigation came into being in any social context was not inquired into. Although medical sociologists in the 1980s and 1990s turned to the social construction of knowledge of disease it remained a minority interest among historians of medicine. Stronger was the compulsion to cultural history, triggered a move to literary and anthropological sources and methodological approaches.

In order to take into stock this new enthusiasm for ‘culture’ and the methodological changes that went with it, one of the doyens of medical history, Charles Rosenberg suggested replacing the category of ‘social context’ with ‘cultural framing’ (Rosenberg 1989, Golden 1992). His suggestion was warmly embraced by many Anglo-American historians of medicine, however, it was predominantly used as a smokescreen to continue what they had done before, namely focussing on the reconstruction of reactions to disease in the past.^6^ By and large, what past societies believed the physiological reality of a disease to be remained unexplored. The task was left to those specialised more in the history of ideas in science and the historical epistemology of natural knowledge.

With regard to the investigation of early modern diseases such as the pox, this was confined mainly to historians who had the necessary expertise in ancient languages and philosophy. Although a small specialised field of study, much interesting literature on early modern conceptions of disease stemmed from it, which took into account the complicated logic and rhetoric of medieval scholasticism and mechanistic philosophies related to the human body emerging in the sixteenth-century (Siraisi 2002, Maclean 2002, Nance 2001). I appreciated these studies because they did not write stories of continuity and origin, but rather underlined the incommensurability of early modern ideas of disease with today’s views, particularly that of specific disease entities.

I was amazed to discern, however, that such works hardly influenced studies on the socio-cultural reaction to early modern disease, and vice-versa; for the most part they simply flourished alongside each other (see for example Wilson 2000). Morever, it became apparent that the most challenging of the suggestions of social constructionism was not discussed, namely that in order to understand disease (past or present) we need to bring together theories of disease and technologies of its treatment with the wider-knowledge generating socio-cultural context in which they are situated. Any understanding of disease is a product of scientific thinking and practice as well as its multiple mediations in a specific socio-cultural space at a given moment in time.

My own work on the pox aimed at bringing together two as yet distinct fields of scholarship with their different ways of ‘re-constructing’ disease in the past. How, I wondered, ought we to understand the socio-cultural responses to the pox in light of sixteenth-century medical discourses on disease causation, symptoms, and signs? I also wanted to know how the bodily experience of the pox and its treatment in a specific social space shaped and structured the intellectual thinking and writing about it. How could we get at, what I came to call, the ‘negotiated identity’ of the sixteenth-century pox? In order to explore these questions, I suggested turning attention to archival and textual materials that might highlight the very moment when disease identity was established in the public domain, that is, to the moment of the diagnostic act itself (Cunningham 2002).

With regard to pox in the fifteenth and sixteenth centuries, it was through the questions that were asked and answered at the time, the decisions made, and the actions that were taken during the act of diagnosis, that we might glimpse the negotiation of its identity. The investigation of the ‘diagnostic act’, I suggested, allowed bringing together the study of medical semiotics (that is the theoretical reflections on the meaning of physical signs) with the various practices in deciphering and treating physical symptoms at the bedside in a specific socio-cultural setting. Let me, by way of example, turn to such ‘diagnostic acts’ in the wards of the civic hospital in Augsburg. From this we can discern some of the potential of the study of the ‘diagnostic acts’ for the understanding of early modern diseases more generally.

In the early hours of a cold January morning in 1618 Philip Ess, accompanied by his wife, presented himself at the municipal pox hospital in Augsburg (Stein 2009). They were convinced that he was suffering from the French pox and had previously sought the advice of different healers. The hospital’s medical team, a barber-surgeon and an academic physician, examined Ess’ carefully in the presence of other witnesses. Ess turned out to be a controversial case. After having discussed his symptoms in great detail, the participants of the examination came to the following verdict: Ess’ signs simultaneously pointed to the French pox AND to a disease that was identified as ‘elefantiasis’. (In medical treatises at the time, the latter was classified as one of the four possible forms of leprosy). Because the signs that pointed to the French pox outnumbered those hinting at leprosy, the medical team decided that a cure in the pox hospital might be beneficial. Ess was then accepted to the male ward. However, only a week later he appears in the hospital record once again. On one of their daily rounds through the wards, his body had attracted the attention of the medical practitioners, and after having kept him under close supervision for a couple of days, they reached the verdict that his signs had morphed into unambiguous signs of leprosy. Their diagnosis triggered immediate consequences for Ess’s institutional fate: considered a health threat to the other hospital inmates, he was immediately dismissed from the pox hospital.

From today’s point of view Philip Ess’s changing diagnosis strikes us as bizarre. But the surviving hospital archival material reveal that his body was only one of many in the civic hospital (and indeed in the two private ones run by the Fugger family) which harboured several diseases simultaneously and whose physical signs of the pox later developed into signs considered to be related to another ailment. In order to explain these phenomena of disease metamorphosis, we must turn to what stands at its core, namely the early modern conception of the physical sign. It is this specific understanding of physical signs, I argue, that allowed for a central characteristic of early modern medicine, its general flexibility and fluidity of disease definition and classification.

Today the term ‘disease’ refers to a pattern of signs that hang together and recur in more or less the same way, in successive individuals. It is only the recurrence of a pattern of events, a number of elements combined in a definite relationship, chronological and geographical, which we label a ‘disease’ (King 1980). A disease consist of a congeries of different signs - no single sign, by itself, makes a ‘disease entity’, such as venereal syphilis, for instance. On this basis contemporary medicine tends to differentiate between subjective ‘symptoms’ that are only felt by the sick individual, and objective ‘signs’, which can be detected by another person (Wear 2000). The other person is usually a physician who is expected to organise the ‘chaos’ of subjective symptoms and to arrange them into a logical, coherent order, associated with a specific disease entity, and described in a medical textbook or visually represented in a medical atlas. Ostensibly, there is no hidden meaning behind the visible sign or symptom; it is transparent to the calculating and authoritative gaze and language of the doctor (Foucault 1973).

Our conception of physical signs of disease would have struck sixteenth-century contemporaries as extremely odd. For them disease and sign were bound together by structure of sensibility incommensurable to ours (Stein 2009). The most striking difference is that physical signs were relatively open to a number of possible meanings conceivably pointing in different directions. A signs was not restricted in its meaning as a signifier of a specific disease entity, but rather, could point to causes that, within the logic of sixteenth-century medical reasoning, were not necessarily related to disease at all. Signs could also point to the present state of the body (natural, non-natural, or even preternatural) at the time of the examination. Further, they possessed a historical dimension and provided clues about past or future physical experiences and possible developments of the disease in question.

The ultimate meaning of how physical sign related to disease had to be gained through the interpretation of its sensible qualities. A crucial consequence of this understanding was that it provided no space for a radical distinction between superior (in terms of truth value) ‘objective’ knowledge, owned by the medical practitioner and the (subordinated ‘subjective’) knowledge felt by the patient. Every diagnosis was inextricably bound up with the disease experience of the ailing individual. As put by one of the very few early modern social historians of medicine to focus on disease construction once put it, ‘the description of the patient’s subjective symptoms of feelings, the patient’s story as it were, were made part of the description of disease…’ (Wear 2000:128). Diagnosing was a complicated and subtle business that relied as much on the experiences, opinions and interpretations of the sufferer as on the professional expertise of the medical practitioner. In fact, the archival material of the pox hospitals in Augsburg revealed that the decisive moment which turned a sign into a sign of the pox was the encounter between the medical practitioners and the applicant in the examination room of the Augsburg civic pox hospital.

The narrative of the sufferer was crucial to making the physical signs ‘speak’. This peculiar understanding of bodily signs, at least from our perspective, was ultimately couched in an epistemology that was based on Aristotelian natural philosophy (Stein 2009). As for Aristotle all knowledge acquisition began with empirical sense experiences, the aim of all knowledge of natural things (including diseases) was to grasp their universal feature through deductive reasoning. In the Aristotelian sense a sensible sign physical therefore was not meaningful in itself but only tentatively pointed to something hidden, the invisible nature or essence of disease. This Aristotelian notion was supported and reinforced by the early-modern idea of a dualistic body, a major symbolic opposition in Western medicine from its first formulation in the ancient Greek Hippocratic treatises. Until far into the eighteenth century the human body was considered a place of hidden and secret activities. Only through the physical signs on the surface of the skin could a sick individual and his or her medical practitioners speculate about the secrets happening inside (Duden 1992).

This understanding was not restricted to learned individuals; it circulated widely among all levels of early modern society (Fissell 2004). The participants in the act of diagnosis at the civic pox hospital, although differing widely in their social and vocational background, shared similar fundamental views of the functioning of the human body to that of medical practitioners – a fact that often led to serious debates over the actual meaning of signs. One example from the civic pox hospital is that of Walburga Reuchart who had brought her three-year old daughter there for an examination (Stein 2009). The girl was a serious and heart-breaking case, her body covered in open lesions and ulcers and she was in terrible pain. However, Doctor Zeller and barber-surgeon Gablinger came to the conclusion that the girl’s signs were not related to the French pox but rather to some kind of poisonous and infectious rash. They therefore refused Reuchart admission and advised her to present her daughter at the Hospital of the Holy Ghost (an odd decision, because this hospital was reserved for old people and strictly refused the admission of suffers with open lesions identified as infectious).

Walburga Reuchart questioned the verdict mainly because she had identified her daughter’s lesions as signs of the pox. Although she was widowed and without any substantial financial means, she managed to obtain the supporting opinion of several healers in town, all whom confirmed her own suspicion, including the medical practitioners at the civic Holy Ghost Hospital. Walburga Reuchart’s conviction of the meaning of her daughter’s signs which, she claimed, had been gained through her own experiences with the pox, is but one example from the hospital records which suggest that sick and their relatives, the medical practitioners in the examination room at the civic hospital in Augsburg all spoke essentially the same language; they were equal partners in a ‘unitarian medical world’ which allowed them to negotiated the meaning of the bodily signs (Jones and Brockliss 1997).

However, the identity of the pox at the pox hospital, I argue, was not only shaped by the negotiation over shared knowledge of the body and the natural world at the moment of the examination. The dynamic of the diagnostic act was also closely linked to the institutional setting in which it took place (Stein 2009). The civic pox hospital in Augsburg was part of a large network of institutions that, for historical, administrative and financial reasons, specialised in treating and caring different diseases and illnesses (leprosy, plague, old age-related ailments, surgical and so on). The definition and differentiation of these different physical conditions involved constant negotiation between the inmates, the appointed medical team and staff, as well as the civic authorities that oversaw the individual institution. In the case of the pox in Augsburg’s civic pox hospital it took almost twenty years of intense and often furious debate between the hospital’s two medical practitioners, with colleagues in rivalling institutions and civic authorities to define what the pox was and fix the respective responsibilities regarding its treatment. Only at the end of such protracted struggles, did the pox diagnosis and its cure come to rest authoritatively with the academic physicians. This triumph cannot be simply understood as rooted in academic credentials; it was also intimately linked to the physicians close links to the city’s ruling elite (the majority married patrician women) that allowed them to influence major decisions in the area of public health. In my study I have shown that their rise to power had immediate important consequence for the ways the pox were conceptualised, diagnosed and treated in the civic hospital.

However, it has to be remembered that physicians’ power over the pox diagnosis could never be absolute. Due to the specific understanding of physical signs, the diagnostic judgement reached during the examination of sick individuals such as Philip Ess or Walburger Ruechart’s daughter was always a merger between the academic physician and the surgeon understanding and agreement of the patient’s condition and the sick’s (or his and her relatives’) perception on the condition. The verdict was a picture of disease that seemed to all the parties involved to be a meaningful reflection of the sick’s condition at a particular moment in time. Most importantly, it was understood by everyone involved that this verdict was not set in stone but flexible and could be altered if the diagnostic circumstances changed.^7^ Diagnostic results were ever open to question and to change, along with the authority of those who had reached them.

What do we gain by investigating the sixteenth-century diseases through the prism of the diagnostic act? Instead of ‘transplanting into the past the hidden or potential existence of the future’, as the sociologists of science Bruno Latour once described the methodology of retrospective diagnosis, the investigation of the diagnostic act itself takes the historicity of the human body and its functioning seriously (Latour 2000:250). What emerges is that the identity of diseases such as the pox was not fixed, but flexible, fluid, temporal and local. By linking both the specific socio-cultural environment in which these diseases occurred and were treated, and the world of early modern medical and philosophical reasoning about this disease and its relationship to complicated and multi-faceted interactions between the human body and the God-created wider world (the so-called micro-macrocosm), we can catch a glimpse at how utterly different the pox was ‘made up’ and that it cannot be identified with our modern disease entity of venereal syphilis. The reconstruction of the diagnostic act estranges the past and thus undermines the idea of an invisible bond between the past and present, which most ‘histories of syphilis’ silently assume. No longer does the present appear as the necessary or inevitable outcome of the past.

## Concluding Remarks: ‘Getting’ the Pox

Why is it important to keep on estranging the past in the history of disease? Why resist the old narratives of origin and continuity? Why not simply admit that the enthusiasm for constructionism, my own included, reflected a specific moment in Western academic thinking of the 1980s and 1990s, which has now passed. And if it is now passé, why not simply return to what most historians of disease have always done well, the socio-cultural reconstruction of the reactions to disease over time?

The reason why I resist is because I believe that it is only through histories of discontinuity that we can maintain to keep a critical distance from the scientific beliefs of the present. It is this distance that permits us to observe and investigate, for example, the current obsession with the neurosciences (that has begun to affect the way historians reconstruct the past).^8^ Foucault’s geneaological approach, which encouraged attending to discontinuities in history, permits us, moreover, to investigate how such obsessions come into being. Historical investigation into the history of disease should be an inquiry both into the grounds of our current knowledge and beliefs about disease (which inspire history writing) and the analytical categories that establish its “inevitability,” that is, understand that the current discussion is itself an interpretation of reality, not reality itself. Historians of disease can contribute to such a history of the present by identifying the sources of current values about disease – how they came into being, the relationships they have constituted, the power they have secured and, most importantly, the actual knowledge/power they have eclipsed or are eclipsing. ‘Getting’ the pox, I’m tempted to say, is no bad thing.

## Footnotes

1 The series was edited by William E. Bynum and his wife Helen. An exception to this tendency is the volume by Sander Gilman on obesity (Gilman 2009).

2 It is probably little wonder that the pox were called ‘Mal de Naples’ in France. In fact, each European country had its own name for it (Bloch 1901).

3 For a detailed discussion of the Fugger family’ endowment of the pox hospitals and the myths surrounding it, see Stein (2009)..

4 One of the few exceptions is the study by Arizzabalaga et al. (1997).

5 In restrospect, it is ironic that it was also the time, when it became first apparent that ‘the state’, particularly in the Anglophone world, was preparing its retreat from its responsibilities regarding health and medical care of its citizens.

6 This is an interesting development because Rosenberg’s suggestion of ‘framing’ was not a simple rejection of the challenge of constructionism. Rosenberg was aware of the provisional nature of knowledge, particularly in regard to disease. He even pointed out that social historians of medicine had ‘failed to focus on the connection between biological event, its perception by patient and practitioner, and the collective effort to make cognitive and policy sense out of this perception’ (Rosenberg and Golden 1992:xvi).

7 For cases in which the changing diagnostic environment produced different diagnostic verdicts in the very same individual, see Stein (2009).

8 For a recent insightful debate on virtues and vices of neurohistory see Isis 105,1 (2014): 100-154.

## References

[R1] Arizzabalaga J, French R, Henderson J (1997). The Great Pox: The French Pox in Early Modern Europe.

[R2] Bloch I (1901). Der Ursprung der Syphilis: Eine medizinhistorische und kulturgeschichtliche Untersuchung. Teil 1: Ursprung der Syphilis.

[R3] Bloch I, Loewenstein G (1912). Prostitution.

[R4] Burckhardt J (1990 [1878]). The Civilization of the Renaissance.

[R5] Cooter R, Huisman F, Warner JH (2006). “Framing” the End of the Social History of Medicine’. Locating Medical History: The Stories and Their Meanings.

[R6] Cooter R, Stein C (2013). Writing History in the Age of Biomedicine.

[R7] Cunningham A (2002). Identifying Disease in the Past: Cutting through the Gordian Knot. Asclepio.

[R8] Duden B (1992). The Women Beneath the Skin: A Doctor’s Patients in Eighteenth-Century Germany.

[R9] Fabricius J (1994). Syphilis in Shakespeare’s England.

[R10] Fissell M (2004). Vernacular Bodies: the Politics of Early Modern England.

[R11] Foucault M (1973 [1963]). The Birth of the Clinic: An Archaeology of Medical Perception.

[R12] Foucault M (1989 [1966]). The Order of Things: An Archaeology of the Human Sciences.

[R13] Gilmann S (2011). Representing Health and Illness: Thoughts for the Twenty-First Century. Medical History.

[R14] Gilman S (2009). Obesity: The Biography.

[R15] Hacking I (2000). The Social Construction of What?.

[R16] Hamlin C (2009). Cholera: The Biography.

[R17] Jackson M (2009). Asthma: The Biography.

[R18] Jones C, Brockliss L (1997). The Medical World of Early Modern France.

[R19] King L, Caplan A (1980). What is Disease?. Concepts of Health and Disease: Interdisciplinary Perspectives.

[R20] Latour B, Daston L (2000). On the Partial Existence of Existing and Nonexisting Objects. Biographies of Scientific Objects.

[R21] Maclean I (2002). Signs and Nature in the Renaissance: The Case of Learned Medicine.

[R22] Nance B (2001). Turquet de Mayerne as Baroque Physician: The Art of Medical Portraiture.

[R23] Quetel C (1992). History of Syphilis.

[R24] Roper L (1991). The Holy Household: Women and Morals in Early Modern Augsburg.

[R25] Rosenberg CE (1989). Disease in History: Frames and Framers. Milbank Quarterly.

[R26] Rosenberg CE, Golden J (1992). Framing Disease: Studies in Cultural History.

[R27] Sauerteig L (1999). Krankheit, Sexualität, Gesellschaft. Geschlechtskrankheiten und Gesundheitspolitik in Deutschland im 19. Und 20. Jahrhundert.

[R28] Schuster P (1992). Das Frauenhaus: Städtische Bordelle in Deutschland, 1350-1600.

[R29] Siraisi N, Kessler E, Maclean I (2002). Disease and Symptom as Problematic Concepts in Renaissance Medicine. Res et Verba in the Renaisssance.

[R30] Stein C (2009). Negotiating the French Pox in Early Modern Europe.

[R31] Tattersall R (2009). Diabetes: The Biography.

[R32] Wear A (2000). Knowledge and Practice in English Medicine, 1550-1680.

[R33] Wilson A (2000). On the History of Disease-Concepts: The Case of Pleurisy. History of Science.

[R34] Wolff J (2012). The Human Right to Health.

